# Characterization of gut microbiota in patients with diabetic kidney disease

**DOI:** 10.3389/fcimb.2026.1713005

**Published:** 2026-02-09

**Authors:** Shoujuan Yu, Huimin Niu, Yan Zhang, Ling Yu, Qi Zhang, Xingchen Liu, Yue Sang, Ran Wang, Min Zhang

**Affiliations:** 1 Department of Nephrology, Affiliated Beijing Chaoyang Hospital of Capital Medical University, Beijing, China; 2 Key Laboratory of Functional Dairy, Co-Constructed by Ministry of Education and Beijing Government, Department of Nutrition and Health, China Agricultural University, Beijing, China; 3 Sports & Medicine Integration Research Center (SMIRC), Capital University of Physical Education and Sports, Beijing, China; 4 Shunyi District Zhangxizhuang Community Healthcare Center, Beijing, China

**Keywords:** correlation analysis, DKD, DM, gut microbiota, microbiota function

## Abstract

**Introduction:**

Diabetic kidney disease (DKD) is a major complication of diabetes mellitus (DM). Although dysbiosis of the gut microbiota in DKD has been reported, the specific microbial species associated with disease progression from DM to DKD remain insufficiently defined.

**Methods:**

We conducted shotgun metagenomic sequencing on fecal samples from 55 healthy participants, 47 patients with DM, and 38 patients with DKD. Gut microbiota diversity, composition, and functional pathways were compared across groups; correlations with glycemic and renal indices were evaluated.

**Results:**

Overall alpha-diversity showed no significantly difference between DKD and healthy controls; however, the simpson’s index was higher in DKD than in DM (p < 0.05). There was a difference in beta-diversity between DKD and the healthy control (p = 0.002), but no significant difference was observed between the DKD and DM group. Bacteria significantly enriched in DM/DKD include *Mediterraneibacter*, *Enterocloster*, *Shigella*, *Limosilactobacillus*, and *Thomasclavelia*, which showed positive correlations with glycemic indicators (HbA1c, fasting blood glucose) and renal indicators (BUN, UACR). In contrast, health-enriched bacteria, *Phocaeicola*, *Faecalibacterium*, *Lachnospira*, *Agathobacter*, *Odoribacter*, and *Paraprevotella* were negatively correlated with these parameters. Functional analysis revealed that compared to the DM group, the DKD group enriched pathways related to aromatic amino acid biosynthesis (phenylalanine, tyrosine, tryptophan), biofilm formation, and lipopolysaccharide biosynthesis. Gut microbial shifts along the DM–DKD correlates with adverse glycemic and renal phenotypes, as well as functional characteristics associated with inflammation and barrier injury. These findings suggest that microbially driven metabolic and structural pathways represent potential targets for mitigating the progression of DKD.

**Conclusion:**

This study elucidates the distinct characteristics of the gut microbiota in DKD patients and highlights potential microbial markers involved in the progression from DM to DKD.

## Introduction

Diabetes mellitus (DM) is a major condition that requires global efforts for its prevention and management. In recent years, the prevalence of diabetes has steadily increased, with approximately 5.29 million new cases reported in 2021 (GBD 2021 Diabetes Collaborators, 2023). By 2050, the number of individuals living with type 2 diabetes is projected to reach 1.31 million (Sun et al., 2022). In China, an estimated 141 million adults are affected, with the country having the highest number of undiagnosed cases worldwide (Xu et al., 2024b). Moreover, DM predisposes individuals to several complications, such as diabetic kidney disease (DKD) and cerebrovascular disease, which significantly compromise prognosis and quality of life (Chong et al., 2024; Niu et al., 2024).

DKD is the most common microvascular complication of type 2 diabetes mellitus, affecting up to 40% of diabetic patients developing DKD (Młynarska et al., 2024a; Wei et al., 2024). Clinically, DKD is characterized by persistent albuminuria and/or a progressive decline in the glomerular filtration rate (GFR) (Gaddy et al., 2025). DKD is the leading cause of end-stage renal disease (ESRD), accounting for approximately 30%-50% of all ESRD cases worldwide (Guo et al., 2024; Jha et al., 2024). Studies have shown that the incidence of severe proteinuria increases with the duration of diabetes, with the cumulative incidence of severe proteinuria reaching 50% after 20 years (Kanasaki et al., 2024). These findings suggest that a longer duration of diabetes increases, the likelihood of developing DKD in affected individuals.

DKD is a common subtype of chronic kidney disease (CKD) (Shen and Su, 2024). A growing body of recent research suggests that disturbances in gut microbial composition and function characterize CKD (Li et al., 2024b). CKD is characterized by reduced α-diversity (lower Shannon and Chao1 indices) and by distinct β-diversity clustering (Voroneanu et al., 2023). This condition is typically characterized by an increased Firmicutes-to-Bacteroides ratio (Tao et al., 2019; Stepanova, 2024). Genus-level analyses revealed significant reductions in key short-chain fatty acid (SCFA) producers (Deng et al., 2024), including *Roseburia*, *Faecalibacterium*, Ruminococcus, and *Lactobacillus johnsonii* (Miao et al., 2024), resulting in decreased SCFA production and compromised intestinal barrier integrity. Concurrently, the accumulation of uremic toxins (e.g., indoxyl sulfate and p-cresol sulfate) and pro-oxidative metabolites triggers metabolic endotoxemia and low-grade systemic inflammation, further exacerbating renal inflammation and fibrogenesis. Together, these finding highlight dysbiosis as a clinically relevant feature of CKD and a potential therapeutic target for CKD treatment.

Similarly, the gut microbiota of patients with DKD is markedly dysbiotic (Mosterd et al., 2021). DKD is associated with microbiome alterations linked to pathways such as metabolite signaling (e.g. indole/kynurenine and bile acids) (Xu et al., 2024a) (Yu et al., 2024), epithelial barrier dysfunction, inflammation, and fibrosis (Chu et al., 2024). Recent reviews on DKD have synthesized these mechanisms and emphasized their translational implications, offering a timely framework for interpreting disease-associated microbial and functional signatures. Kim et al. reported that bacterial taxa such as *Alistipes onderdonkii*, *Ruminococcus*, and *Bacteroides intestinalis* were significantly enriched in the gut of patients (Kim et al., 2023). These findings suggest that the gut microbiota undergo specific alterations in DKD. However, the precise role of the gut microbiota in DM pathogenesis and the underlying regulatory mechanisms remain largely unexplored. Building on this literature, our study compared metagenomic profiles across Healthy, DM, and DKD cohorts to identify taxa and pathways associated with DKD progression.

Therefore, this study collected fecal samples from subjects with DM, DKD, and healthy controls. Metagenomic sequencing technology was employed to analyze the composition and function of the gut microbiota. The aim of this research is to investigate pathogenic bacteria playing a pivotal role in the progression from DM to DKD and elucidate the association between the gut microbiota and diabetic complications.

## Materials and methods

### Inclusion and exclusion criteria

DM was diagnosed according to the criteria set forth by the American Diabetes Association’s Standards of Care in Diabetes-2025 (Elsayed et al., 2025). Specifically, a diagnosis of diabetes is made when a patient exhibits typical symptoms of diabetes (such as polyphagia, polydipsia, polyuria, and unexplained weight loss) in conjunction with one of the following. A random blood glucose level greater than or equal to 11.1 mmol/L; a fasting plasma glucose level greater than or equal to 7.0 mmol/L; a 2-hour post-glucose tolerance test glucose level of 11.1 mmol/L or higher; or an HbA1c level greater than or equal to 6.5%. DKD is diagnosed when diabetes is confirmed as the cause of renal damage, and other causes of chronic kidney disease are excluded. Provided at least one of the following criteria is met: (1) In the absence of interfering factors, the urinary albumin-to-creatinine ratio (UACR) exceeds 30 mg/g in at least two out of three tests conducted within 3 to 6 months, or the 24-hour urinary albumin excretion rate exceeds 30 mg/24h. (2) The estimated glomerular filtration rate (eGFR), is less than 60 mL/min/1.73m², sustained for more than 3 months. (3) Kidney biopsy reveals pathological changes consistent with diabetic nephropathy (Tan et al., 2025a). DKD and DM volunteers were recruited through rigorous screening by physicians in the departments of nephrology and endocrinology. All enrolled participants were newly diagnosed with the disease and had not taken any related medications.

### Participants recruitment

This was a clinical epidemiological study conducted at Beijing Chaoyang Hospital. A total of 140 participants participated in this study, including 55 healthy controls (Healthy), 47 patients with type 2 diabetes mellitus (DM), and 38 patients with diabetic kidney disease (DKD). All participants were recruited from Beijing Chaoyang Hospital of Capital Medical University and provided written informed consent prior to the clinical observational study. The study was approved by the Ethics Committee of the Affiliated Beijing Chaoyang Hospital of Capital Medical University (ethical approval number 2024-Section-2-3). This study has been registered with the China Clinical Trials Registry under the registration number ChiCTR2400086609.

### Metagenomic sequencing

Feces samples are collected in sterile stool collection tubes. All samples are stored at -80°C after collection (Salguero et al., 2019). Total DNA was extracted from the participants fecal samples using a DNA extraction kit (QIAGEN, Hilden, Germany). The concentration and quality of the DNA were assessed using NanoDrop 2000 spectrophotometer (Thermo Scientific, Waltham, USA) and 1% agarose gel electrophoresis. Fecal metagenomic analysis was performed using shotgun metagenomic sequencing on the Illumina NovaSeq PE150 platform (Illumina Inc., San Diego, USA) at Majorbio Bio-Pharm Technology Co., Ltd. (Majorbio, Shanghai, China) (Zhang et al., 2024).

### Metagenomic analysis

The quality of raw sequencing data was initially assessed using Fastp to evaluate key metrics such as base quality distribution, GC content, and splicing contamination (Bouras et al., 2023). Subsequently, trimmomatic was applied to remove low-quality bases, junction sequences, and other contaminant sequences (Tang et al., 2024). Human genomic sequences were then filtered out using Bowtie2 software. Stringent host-read removal as above; *post-hoc* frequency-based contaminant screening (library input as proxy), removing ultra-low-prevalence/abundance features typical of reagent contaminants; and sensitivity analyses excluding batch-specific signals. Sequence data, post-QC, were subjected to multi-mix splicing using Megahit software. Alpha diversity (e.g., Shannon index, Chao1 index) and beta diversity (e.g., PCA, PCoA) were calculated using QIIME. Functional annotations based on the Kyoto Encyclopedia of Genes and Genomes (KEGG) were assigned to the non-redundant gene set. Differential analyses were conducted at the taxonomic, functional, and gene levels using the Wilcoxon rank-sum test, Kruskal–Wallis test, and LEfSe analysis. Differences were compared at the taxonomic, functional, and gene levels.

### Statistical analysis

GraphPad Prism (Version 8, GraphPad Prism Institute, Inc., San Diego, California, USA) and SPSS 20 software (SPSS Inc., Chicago, IL, USA) were used to analyze data. Wilcoxon rank-sum test, Kruskal–Wallis analysis was used to test for differences between mean values. A p < 0.05 value indicated statistical significance.

## Results

### The baseline information of participants

**Table 1** presents clinical information of the participants, including age, gender, body mass index (BMI), hemoglobin A1c (HbA1c), fasting blood glucose, fasting insulin, serum creatinine, blood urea nitrogen (BUN), estimated glomerular filtration rate (eGFR), urinary albumin-to-creatinine ratio (UACR), acute C-reactive protein (CRP), and 25-hydroxyvitamin D3 (25(OH)VitD3). The baseline information of the DM and DKD groups was generally matched.

**Table 1:** Baseline clinical indexes of participants.

Characteristics	DM (n=47)	DKD (n=38)	Healthy (n=55)	P_1_	P_2_
Age, years	62.6 ± 9.5	61.6 ± 10.0	35.4 ± 5.0	<0.01	>0.05
Gender, Male n (%): Female n (%)	29 (61.7%):18 (38.3%)	23(60.5%):15(39.5%)	34(61.8%):21(38.2)	>0.05	>0.05
BMI, kg/m^2^	25.2 ± 2.9	24.5 ± 2.6	23.5 ± 2.8	0.01	>0.05
HbA1c	9.08 ± 2.30	7.59 ± 1.59	5.63 ± 0.43	<0.01	<0.01
Fasting Blood Glucose (mmol/L)	8.38 ± 3.01	7.88 ± 2.22	4.43 ± 0.64	<0.01	>0.05
Fasting Insulin (μIU/ml)	8.34 ± 8.04	6.91 ± 13.78	2.33 ± 1.75	<0.01	>0.05
Serum creatinine (μmol/L)	71.73 ± 20.58	137.97 ± 220.01	64.39 ± 14.7	<0.01	0.04
BUN (mmol/L)	6.49 ± 1.55	7.65 ± 4.39	4.75 ± 1.04	<0.01	>0.05
eGFR	87.12 ± 27.45	86.53 ± 38.94	107.33 ± 26.19	<0.01	>0.05
UACR	47.24 ± 201.04	723.86± 1050.72	14.53± 4.81	<0.01	<0.01
CRP	2.11 ± 1.98	5.03 ± 4.59	3.5 ± 1.37	<0.01	<0.01
25(OH)VitD3	17.5 ± 4.3	17.02 ± 4.76	21.43 ± 11.68	0.01	>0.05

Data are presented as mean ± standard deviation, n (%). P_1_ denotes the overall comparison across Healthy, DM, and DKD groups; P_2_ denotes the DM vs DKD groups comparison.

### Alpha diversity of gut microbiota in patients with DKD

The fecal microbiome was analyzed using metagenomic shotgun sequencing, which detected a total of 45,563,103 genes, with an average gene length of 653.94 bp. The number of cataloged genes was 3,811,074, with an average gene length of 726.61 bp. Alpha diversity analysis was used to assess the richness and diversity of microbial communities/functions in environmental samples, employing various diversity indices. In this study, we used the Shannon, Simpson, Chao, Ace, and Sob indices to explore the alpha diversity of the intestinal microbiota in patients with DKD. The Shannon and Simpson indices reflect community diversity, while the Chao, Ace, and Sob indices are indicative of community richness. Notably, higher Shannon index values indicate greater diversity, whereas the Simpson index shows the opposite trend, with higher values corresponding to lower diversity.

**Figure 1:**
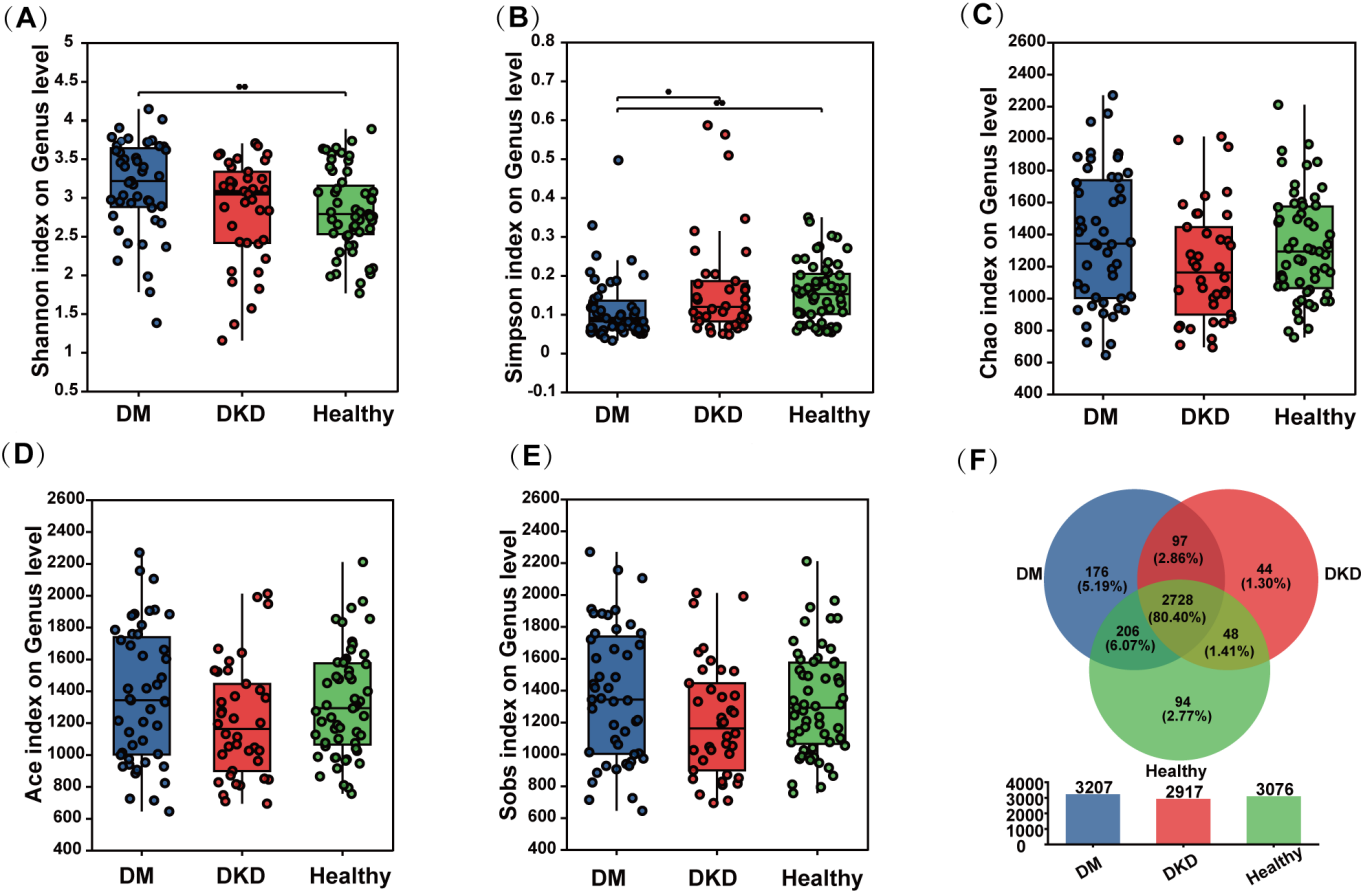
Alpha-Diversity of gut microbiota in different participants groups. **(A)** Shannon index of gut microbiota in the DM, DKD, and Healthy groups **(B)** Simpson index **(C)** Chao index of gut microbiota in the DM, DKD, and Healthy groups **(D)** Ace index of gut microbiota in the DM, DKD, and Healthy groups **(E)** Sobs index of gut microbiota in the DM, DKD, and Healthy groups **(F)** Veen plot of gut microbiota in the DM, DKD, and Healthy groups. DM, Type 2 diabetes mellitus patients; DKD, diabetic kidney patients; Healthy, healthy controls. *P < 0.05, **P < 0.01.

As shown in **Figure 1A**, the Shannon diversity index was significantly higher in both DKD and DM patients compared to healthy controls, with a significant difference observed between DKD and healthy controls (p < 0.01). Notably, the Simpson index was significantly higher in both healthy and DKD patients than in DM patients (p < 0.05), with no significant difference between healthy and DKD patients. This resulted in the diversity ranking of the three groups as DM > DKD > Healthy (**Figure 1B**). Additionally, no significant differences were observed in the Chao, Ace, and Sob indices across the three groups, suggesting similar community richness among the participants in each group (**Figures 1C-D**). The result in **Figure 1E** shows that the total number of species shared by the three groups (DKD, DM, and Healthy) was 2,728, representing 80.40% of the total species. This result shows that the amount of microbiota similar between patients and healthy individuals.

### Beta diversity of gut microbiota in patients with DKD

**Figure 2:**
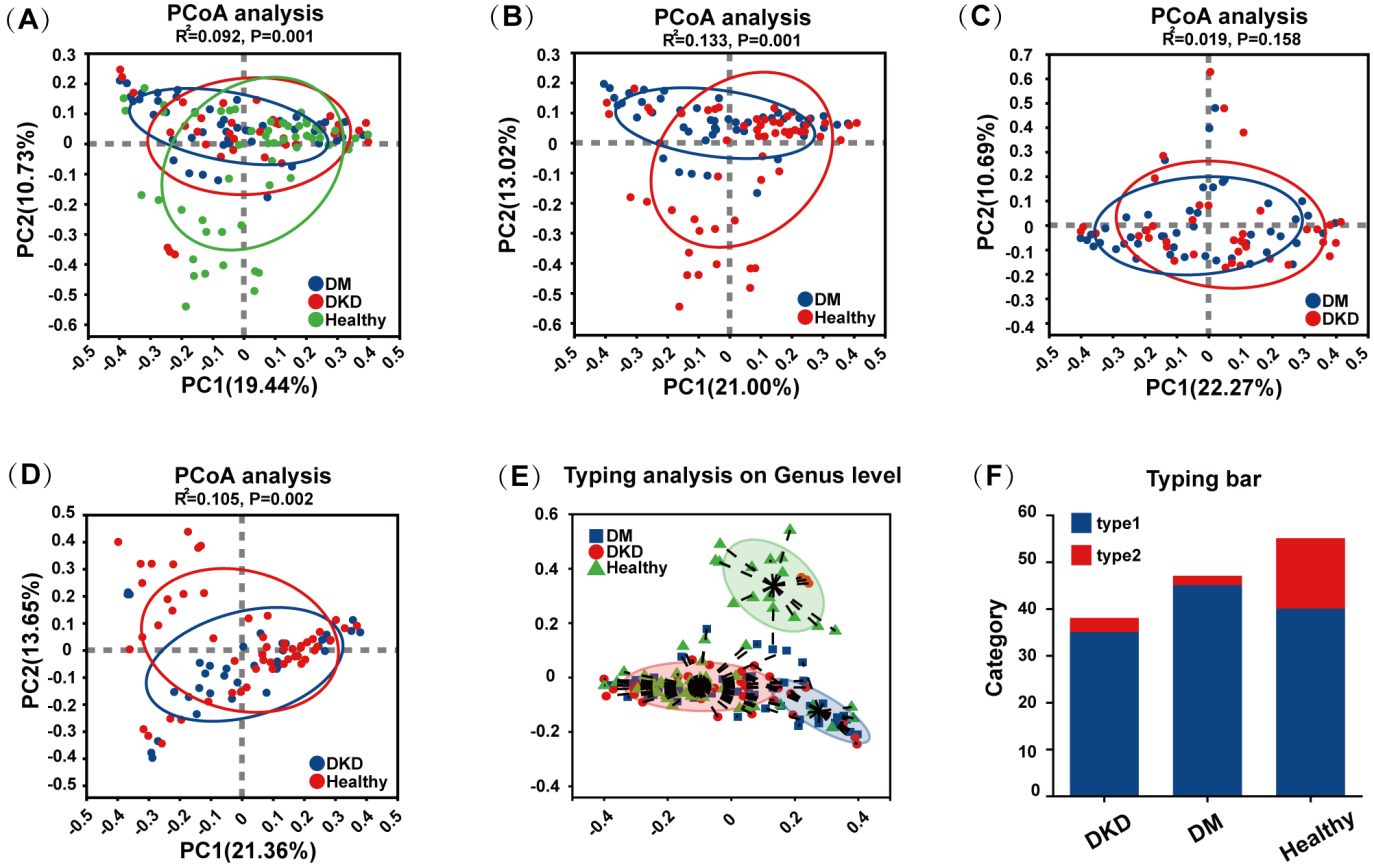
Beta-diversity of gut microbiota in different participants groups. **(A)** Beta -diversity analysis of gut microbiota in the DM, DKD, and Healthy groups **(B)** Beta -diversity analysis of gut microbiota in the DM and Healthy groups **(C)** Beta -diversity analysis of gut microbiota in the DM and DKD groups **(D)** Beta -diversity analysis of gut microbiota in the DKD and Healthy groups **(E)** Genus-level typing analysis among different groups **(F)** Colony typing bar in different groups.

Beta diversity was assessed using Principal Coordinates Analysis (PCoA). Analysis based on Bray-Curtis distances combined with the ANOSIM test revealed a significant separation in microbial community composition among DM, DKD, and healthy controls (**Figure 2A**, ANOSIM, R^2^ = 0.092, p = 0.001). **Figures 2B–D** demonstrate significant differences in microbial diversity among the DM, DKD, and healthy groups, but no significant differences exist between the DM and DKD groups (ANOSIM, R^2^ = 0.019, p = 0.158). Additionally, **Figures 2E, F** indicate that the healthy group exhibited a distinct.

enterotype compared to the DM and DKD groups; specifically, the DM and DKD groups were predominantly characterized by Type 1 bacteria with a smaller proportion of Type 2 bacteria, whereas the healthy group had a higher percentage of Type 2 bacteria.

### Gut microbiota composition and differential analysis

**Figure 3:**
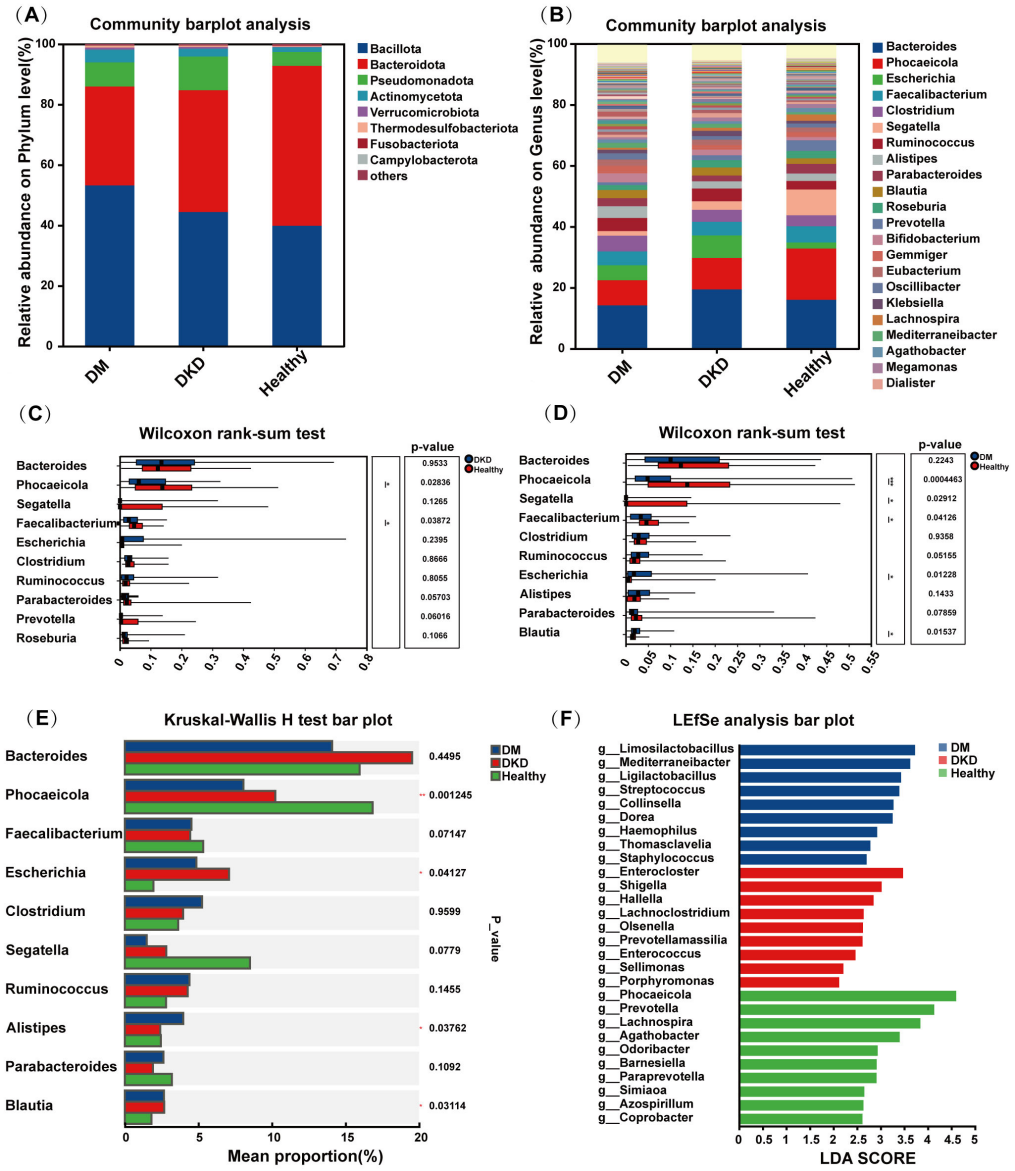
Gut microbiota composition and analysis of variance. **(A)** Composition of gut microbiota at phylum level **(B)** Composition of gut microbiota at genus level **(C)** Results of Wilcoxon rank-sum test for gut microbiota in DKD and Healthy groups **(D)** Results of Wilcoxon rank-sum test for gut microbiota in DM and Healthy groups **(E)** Results of Kruskal-Wallis H test for three groups **(F)** Results of LEfSe analysis in the DM, DKD, and Healthy groups.

To compare the gut microbiota between DKD and DM patients, we analyzed the composition of the microbial communities across different groups. As shown in **Figure 3A**, at the phylum level, the predominant taxa include *Bacteroidota*, *Bacillota*, *Pseudomonadota*, *Actinomycetota*, *Thermodesulfobacteriota*, *Verrucomicrobiota*, *Fusobacteriota*, and *Campylobacterota*. At the genus level, the major genera identified were *Phocaeicola*, *Bacteroides*, *Escherichia*, *Segatella*, *Faecalibacterium*, *Parabacteroides*, *Roseburia*, and *Bifidobacterium* (**Figure 3B**).

Subsequently, we compared the gut microbial communities of DKD, DM patients, and healthy controls using the Wilcoxon rank-sum test. Compared with healthy controls, DKD patients exhibited an enrichment of *Bacteroides*, *Segatella*, *Escherichia*, *Clostridium*, and *Ruminococcus*, while DM patients showed increases in *Clostridium*, *Ruminococcus*, and *Escherichia coli* (**Figures 3C, D**). Further, using the Kruskal-Wallis H test and LEfSe analyses, we identified significant differences bacteria among the three groups. The Kruskal-Wallis H test revealed that the abundances of *Phocaeicola*, *Escherichia*, *Alistipes*, and *Parabacteroides* significantly differed among the groups (**Figure 3E**). LEfSe analysis revealed significant enrichment of *Limosilactobacillus* and *Mediterraneiibacter* in the DM group, *Enterocloster*, *Shigella* in the DKD group, and *Phocaeicola*, *Prevotella*, and *Lachnospira* in the healthy group.

Our comparative analysis indicates that *Mediterraneibacter*, *Enterocloster*, *Shigella*, *Limosilactobacillus*, *Thomasclavelia*, *Lachnoclostridium*, *Hallella*, *Eggerthella*, *Sellimonas*, and *Staphylococcus* were simultaneously enriched in both patient groups (**Table 2**). Conversely, several bacterial taxa were significantly depleted in patients with DM and DKD, primarily including *Phocaeicola*, *Faecalibacterium*, *Lachnospira*, *Agathobacter*, *Odoribacter*, *Paraprevotella*, *Hominiventricola*, *Coprobacter*, *Simiaoa*, and *Azospirillum*. Further research is needed to investigate their potential role in disease progression (**Table 3**).

**Table 2:** Bacteria significantly enriched in both DKD and DM groups compared to healthy controls.

Name	DM Mean(%)	DM-Sd(%)	DKD-Mean(%)	DKD-Sd(%)	Healthy-Mean(%)	Healthy-Sd(%)	Pvalue(DMvsHealthy)	Pvalue(DKDvsHealthy)
Mediterraneibacter	1.50E+00	2.32E+00	1.20E+00	1.13E+00	6.94E-01	5.21E-01	4.14E-04	5.33E-03
Enterocloster	8.80E-01	9.61E-01	1.03E+00	1.92E+00	4.82E-01	7.89E-01	4.42E-03	1.17E-02
Shigella	2.15E-01	3.70E-01	3.16E-01	6.37E-01	7.26E-02	1.65E-01	1.16E-03	4.26E-02
Limosilactobacillus	8.73E-01	4.60E+00	2.90E-01	1.13E+00	1.59E-02	5.20E-02	5.49E-06	1.94E-03
Thomasclavelia	3.17E-01	3.29E-01	2.82E-01	2.58E-01	2.08E-01	2.22E-01	1.19E-03	3.01E-02
Lachnoclostridium	2.29E-01	2.04E-01	2.58E-01	2.20E-01	1.65E-01	1.91E-01	4.19E-02	1.56E-02
Hallella	2.56E-02	1.40E-01	1.40E-01	8.08E-01	2.49E-02	4.07E-02	1.54E-05	4.18E-03
Eggerthella	9.20E-02	1.05E-01	5.99E-02	8.94E-02	3.03E-02	3.72E-02	1.06E-05	1.66E-02
Sellimonas	2.99E-02	5.85E-02	5.36E-02	2.04E-01	1.54E-02	2.92E-02	1.43E-03	2.73E-02
Staphylococcus	1.45E-01	7.03E-01	4.03E-02	4.86E-02	3.74E-02	1.08E-01	1.66E-04	5.33E-03

**Table 3:** Bacteria significantly depleted in both DKD and DM groups compared to healthy controls.

Name	DM-Mean(%)	DM-Sd(%)	DKD-Mean(%)	DKD-Sd(%)	Healthy-Mean(%)	Healthy-Sd(%)	Pvalue(DMvsHealthy)	Pvalue(DKDvsHealthy)
Phocaeicola	7.76E+00	8.81E+00	9.91E+00	9.22E+00	1.63E+01	1.35E+01	4.46E-04	2.84E-02
Faecalibacterium	4.24E+00	4.03E+00	4.25E+00	4.33E+00	5.10E+00	3.10E+00	4.13E-02	3.87E-02
Lachnospira	6.37E-01	9.71E-01	9.85E-01	1.99E+00	2.00E+00	1.89E+00	6.32E-07	4.15E-05
Agathobacter	1.06E+00	1.62E+00	8.17E-01	1.31E+00	1.34E+00	1.41E+00	3.68E-02	4.96E-03
Odoribacter	2.43E-01	2.22E-01	2.72E-01	4.04E-01	4.15E-01	3.55E-01	1.25E-02	5.87E-03
Paraprevotella	2.21E-01	6.21E-01	2.24E-01	5.05E-01	3.72E-01	4.81E-01	2.56E-05	8.83E-04
Hominiventricola	3.25E-02	3.36E-02	3.81E-02	5.08E-02	7.33E-02	7.57E-02	2.16E-04	1.45E-03
Coprobacter	7.86E-02	1.31E-01	7.73E-02	1.04E-01	1.56E-01	1.64E-01	3.74E-04	7.24E-04
Simiaoa	6.81E-02	1.72E-01	6.67E-02	2.73E-01	1.38E-01	3.83E-01	4.26E-02	4.61E-03
Azospirillum	1.58E-02	4.84E-02	7.40E-02	3.66E-01	1.09E-01	3.53E-01	1.25E-02	4.50E-02

### Analysis of functional differences in gut microbiota

**Figure 4:**
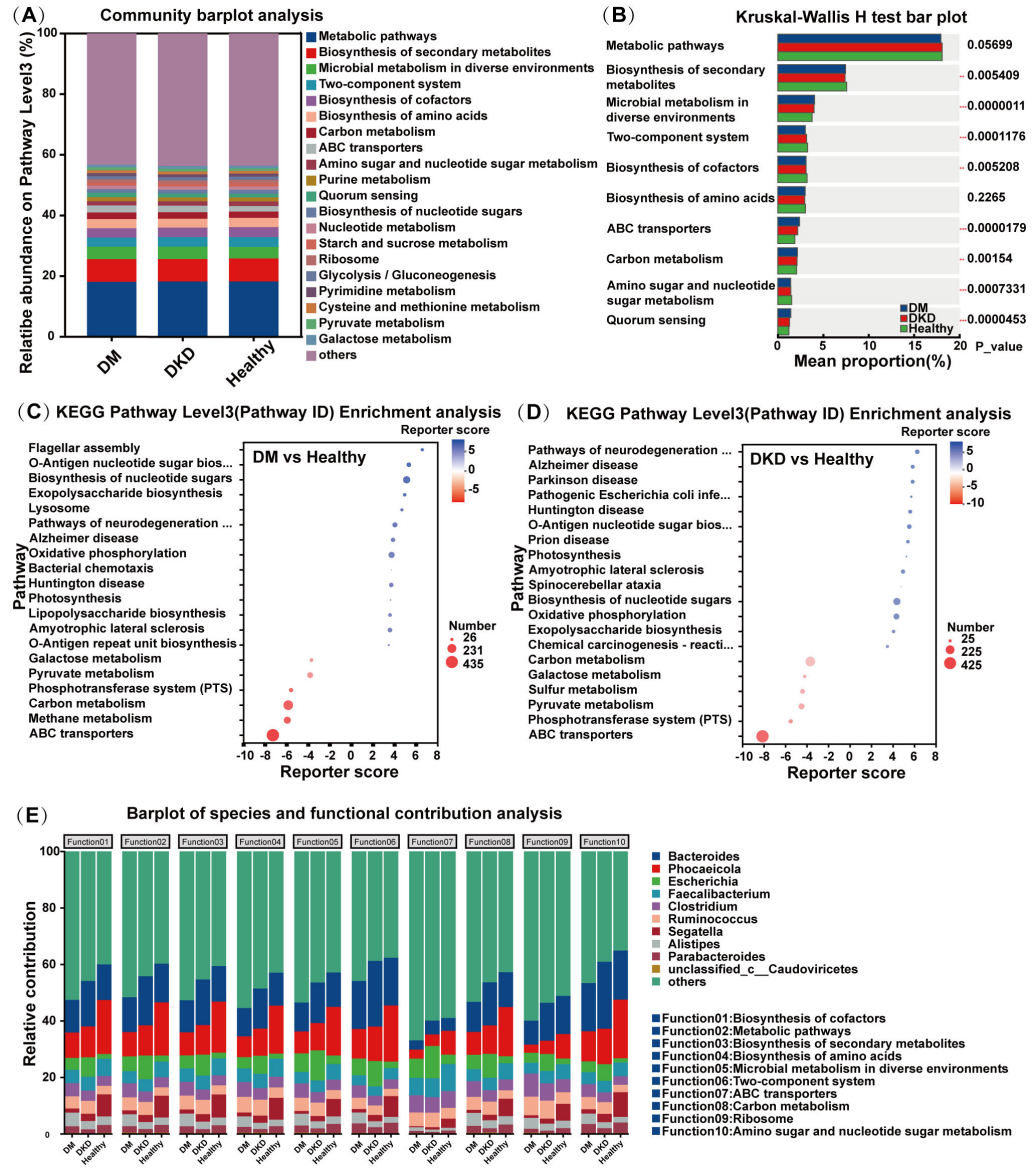
Functional analysis of gut microbiota in different participants groups. **(A)** Relative abundance on KEGG pathway Level3 bar plot in the DM, DKD, and Healthy groups **(B)** KEGG pathway Kruskal-Wallis H test analysis in the DM, DKD, and Healthy groups **(C)** DM and Healthy group KEGG pathway enrichment analysis **(D)** DKD and Healthy group KEGG pathway enrichment analysis **(E)** Barplot of species and functional contribution analysis in the DM, DKD, and Healthy groups.

We analyzed the functional profiles of the fecal microbiota from different volunteer groups using the KEGG database. At KEGG pathway level 3, the predominant pathways across all groups included metabolic pathways, biosynthesis of secondary metabolites, microbial metabolism in diverse environments, two-component systems, and biosynthesis of cofactors (**Figure 4A**). Multivariate analysis revealed significant differences among groups in pathways including the biosynthesis of secondary metabolites, microbial metabolism in diverse environments, two-component systems, biosynthesis of cofactors, ABC transporters, carbon metabolism, amino sugar, and nucleotide sugar metabolism. (**Figure 4B**). KEGG level 3 enrichment analysis demonstrated that DKD patients exhibited significantly higher enrichment in pathways associated with galactose metabolism, pyruvate metabolism, the phosphotransferase system (PTS), carbon metabolism, methane metabolism, and ABC transporters compared to healthy controls (**Figure 4C**). Compared with the healthy control group, pathways such as carbon metabolism, galactose metabolism, sulfur metabolism, pyruvate metabolism, the phosphotransferase system (PTS), and ABC transporters were significantly enriched in the DM group (**Figure 4D**). Compared with the DM group, the DKD patient group exhibited significant enrichment in bacterial chemotaxis, phenylalanine, tyrosine and tryptophan biosynthesis, *Escherichia coli* biofilm formation, flagellar assembly, and lipopolysaccharide biosynthesis (**Supplementary Figure S1**). Additionally, **Figure 4E** presents species distribution and functional contribution analysis, highlighting the significant contributions of *Bacteroides*, *Phocaeicola*, and *Escherichia* to various functional pathways.

### Differential gut microbiota and clinical phenotype correlation analysis

**Figure 5:**
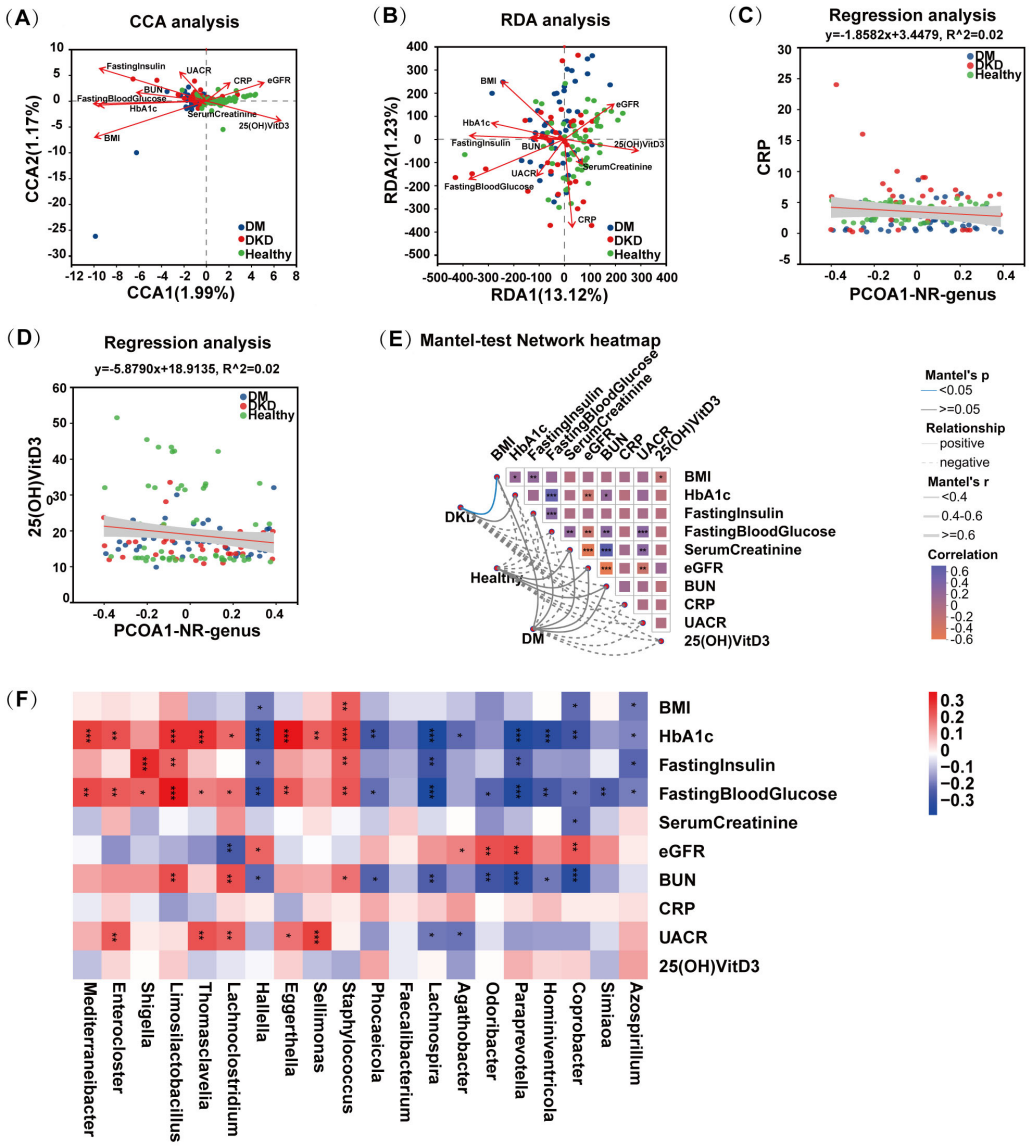
Differential gut microbiota and clinical phenotype correlation analysis. **(A)** CCA analysis of gut microbiota and clinical phenotypes **(B)** RDA analysis of gut microbiota and clinical phenotypes **(C)** Linear Regression analysis of CRP and gut microbiota structure **(D)** Linear Regression analysis of 25(OH)VitD_3_ and gut microbiota **(E)** Mantel-test Network Heatmap **(F)** Spearman Correlation analysis of significantly enriched and depleted bacteria in the gut of DM and DKD patients with clinical phenotype. *P < 0.05, **P < 0.01, ***P < 0.001,.

Numerous clinical phenotypes can affect the composition of the gut microbiota; however, many of these factors exhibit strong multicollinearity, which may confound subsequent correlation analyses. To mitigate this issue, we first screened the clinical phenotypes and found that the variance inflation factor (VIF) values for the 10 clinical phenotypes were all below 10, indicating minimal multicollinearity and supporting their use in further analyses. We then performed canonical correspondence analysis (CCA) and redundancy analysis (RDA) to assess the relationships between clinical phenotypes and both bacterial community structure and function. As shown in **Figure 5A**, the longer arrow lengths for fasting insulin, fasting blood glucose, HbA1c, BMI, 25(OH)VitD_3_, and eGFR indicate a stronger influence on the gut microbiota. The acute angle observed between 25(OH)VitD_3_, eGFR, and healthy controls indicates a positive correlation between these indicators and the microbiota of healthy controls. Conversely, the acute angles between fasting insulin, fasting blood glucose, HbA1c, and BMI with patient groups indicates that these parameters are positively correlated with the gut microbiota of patients with DM and DKD. Similarly, **Figure 5B** shows that the acute angle between 25(OH)VitD_3_, eGFR, and healthy controls indicates a positive correlation with functional characteristics of the gut microbiota.

To assess the impact of clinical factors on the beta diversity of the gut microbiota, we performed linear regression analysis (**Figures 5C, D**). The analysis revealed that CRP and 25(OH)VitD_3_ were negatively correlated with beta diversity. Additionally, a Mantel-test network heatmap was generated to examine the relationship between the distance matrices of the microbiota and clinical phenotypic variables. As shown in **Figure 5E**, the microbiota of DKD patients exhibited a significant positive correlation with BMI, and the microbiota showed significant positive correlations with BMI, HbA1c, and fasting insulin. Conversely, in healthy controls, the microbiota showed negative correlations with BMI, HbA1c, and fasting insulin.

Subsequently, we focused on the correlations between clinical phenotypes and the top ten most significantly enriched and depleted bacterial taxa in DKD and DM patients. **Figure 5F** showed the results of Spearman correlation analysis. The 10 bacteria on the left are significantly enriched in DKD and DM patients, while the 10 bacteria on the right are enriched in healthy controls. Analysis indicated that bacteria enriched in patients were positively correlated with most clinical indicators, particularly BMI, HbA1c, and UACR. Bacteria enriched in healthy controls were predominantly negatively correlated with these clinical parameters.

## Discussion

DKD is a form of chronic kidney disease resulting from diabetes mellitus and characterized by persistent albuminuria and/or decreased glomerular filtration rate (GFR). It is a common microvascular complication in patients with DM. Some studies have reported that up to 40% of individuals with diabetes develop DKD, making it the leading cause of end-stage renal disease (Scilletta et al., 2023). Hyperglycemia is the primary factor responsible for diabetes-specific lesions in the glomeruli and proximal tubules of the kidney. The accumulation of oxidative byproducts induced by hyperglycemia is believed to be one of the major mechanisms that activate diabetic vasculopathic pathways, leading to cellular redox imbalances and oxidative stress (Jung and Yoo, 2022). Numerous studies have highlighted that dysbiosis of the gut microbiota is closely associated with diabetes, nephropathy, and other related conditions. Research on the gut-kidney axis has demonstrated that the gut microbiota affects gut barrier function, renal metabolism, inflammation, and homeostasis of the immune microenvironment, thereby influencing the onset and progression of DKD (Cao et al., 2022). However, the specific gut microbiota involved in the progression from DM to DKD remains to be elucidated. Therefore, this study aimed to investigate the differences in gut microbiota composition between patients with diabetic nephropathy, those with diabetes, and healthy individuals, to identify key microbial species associated with the progression of DM to DKD.

Body Mass Index (BMI) serves as an indicator of obesity, with a BMI over 24 classified as overweight. In this study, both patients with DKD and DM patients had BMI values exceeding 24, indicating that both groups were overweight. This aligns with the findings of Wang et al., who noted that weight-adjusted waist circumference, another distinct indicator of obesity, is associated with DKD in T2DM patients (Wang et al., 2024). Hemoglobin A1c (HbA1c) reflects the average blood glucose level over the past 8–12 weeks and is widely used as a marker for diabetes management; a value above 6.5%, along with typical diabetic symptoms, is diagnostic for diabetes. Our findings indicated that HbA1c levels in the DM and DKD groups were ≥ 6.5%, whereas the levels in the healthy controls group remained within the normal range. Patients with DM and DKD had blood glucose and fasting insulin levels exceeding these ranges. Serum creatinine, blood urea nitrogen (BUN), estimated glomerular filtration rate (eGFR), and urinary albumin-to-creatinine ratio (UACR) are indicators of renal function. The normal ranges for these parameters are 57–97 μmol/L for serum creatinine, 3.1–8.0 mmol/L for BUN, eGFR ≥ 90 mL/min/1.73 m², and UACR < 30. The results indicated that, that the kidney function parameters in patients with DM were normal. In contrast, the renal indices of patients with DKD exhibited abnormal values for all indices except BUN. Additionally, C-reactive protein (CRP) levels, with a normal range of 3–10, were within the normal limits for all groups. In contrast, the levels of 25(OH)VitD_3_, typically normally range from 30 to 100, were below the normal range in all three groups.

Recent research on the diversity and composition of gut microbiota in individuals with DM, DKD, and healthy populations has produced inconsistent findings. In the present study, we observed that the Shannon index was significantly higher, and the Simpson index markedly lower, in the gut microbiota of patients with DM and DKD than healthy controls, suggesting that the diversity of the microbiota was greater in patients with DM and DKD than in healthy controls. Some studies have reported lower microbiota diversity in patients with DKD than healthy individuals (Li et al., 2024a), whereas others have found no significant differences in diversity between these groups (Tao et al., 2019; Gurung et al., 2020; Du et al., 2021). The literature on this topic remains inconsistent, necessitating further research to needed to explore the alpha diversity of gut microbiota in patients with DKD. Beta diversity analysis revealed no significant differences between the microbiota of patients with DKD and those with DM. However, important differences emerged when comparing these patients to healthy controls. This suggests that while the microbiota of DKD and DM patients may be similar, they differ notably from that of healthy individuals. Zhang et al. also found no significant difference in beta diversity between healthy controls and patients with DKD (Zhang et al., 2022), which could be attributed to variations in sample size and geographic location.

The balance between gut symbionts and pathogenic bacteria is crucial for maintaining intestinal barrier integrity and normal renal function. In patients with DKD, this balance is disrupted, leading to a loss of intestinal barrier integrity, activation of immune cells, and secretion of pro-inflammatory cytokines, which further exacerbate kidney dysfunction and the gut microbiota-host relationship. One study identified guild-level microbiome signatures that outperform low-resolution taxonomic markers, suggesting the presence of coordinated functional consortia in disease (Tang et al., 2024). Contemporary reviews have integrated barrier disruption, inflammation/fibrosis, and metabolite pathways (indole/kynurenine, LPS and biofilm) as convergent mechanisms in DKD (Chu et al., 2024). Complementing association data, mendelian randomization now supports a causal contribution of the gut microbiome to CKD susceptibility and kidney function traits (Tan et al., 2025b). Clinically, gut-derived toxins such as TMAO are associated with adverse outcomes in CKD. Indoxyl sulfate lowering with AST-120 has shown biological and preliminary clinical signals, underscoring tractable targets along the microbiome–host axis of CKD (Cheng et al., 2025). Interventional studies including probiotic/synbiotic and soluble fiber trials, have reported reductions in inflammatory markers or uremic toxins, albeit with heterogeneous effects on renal endpoints, highlighting both the promise and the need for longer DKD-specific trials (Cui et al., 2024; Liu et al., 2024a).

Previous studies have identified variations differences in the gut microbiota composition between individuals with type 2 diabetes mellitus and related complications and healthy controls. The primary dysbiotic alterations in these populations include an increase in opportunistic pathogenic bacteria and a decrease in beneficial bacteria, such as SCFA-producing microbiota (for example, *Faecalibacterium* and *Roseburia*) (Huang et al., 2024; Wu et al., 2025). Specifically, the levels of four *Lactobacillus* species were elevated, whereas the abundance of five *Clostridium* species was reduced in individuals with T2DM (Młynarska et al., 2024b). This study observed that *Phocaeicola* and *Faecalibacterium* were significantly depleted in patients with DKD and DM, whereas *Escherichia* species were enriched in these patients. *Faecalibacterium* and *Phocaeicola* are the major butyrate producers in healthy controls. Possesses anti-inflammatory properties, maintains bacterial enzyme activity, and protect the digestive system from intestinal pathogens (Gindt et al., 2024; Price et al., 2024). These bacteria can ferment to produce SCFAs in the human gut, which are widely recognized for their crucial role in maintaining human health (Singh et al., 2022). SCFAs serve as essential nutrients and energy sources for intestinal epithelial cells, protect the intestinal mucosal barrier, reduce inflammation, and enhance gastrointestinal motility. Additionally, these microbes are involved in synthesizing vitamins and other bioactive compounds.

Our study focused on bacteria that are significantly enriched in patients with DKD and DM. **Tables 2** and **3** enumerate the top ten differentially abundant bacteria, including *Enterocloster*, *Shigella*, *Lachnoclostridium*, *Hallella*, and *Sellimonas*, which were enriched in both DKD and DM patients, with a higher abundance in the DKD cohort than in the DM cohort. Previous studies have documented that *Enterocloster* can proliferate extensively in antibiotic-treated mice, thereby diminishing the efficacy of immunotherapy for inflammatory bowel disease. *Shigella*, a genus of gram-negative, short rods is the most prevalent pathogen responsible for bacterial dysentery in humans (Thingholm et al., 2019; Hu et al., 2020). *Lachnoclostridium*, a gram-positive, obligate anaerobic, spore-forming, and motile bacterium, has been shown to reduce acetate levels in circulation, potentially contributing to increased abdominal fat and adversely affecting obesity and type 2 diabetes (Dandachi et al., 2021; Liu et al., 2024b). The abundance of *Lachnoclostridium* is significantly elevated in pregnant women with gestational diabetes (Hu et al., 2023). Furthermore, the relative abundance of *Lachnoclostridium* varies across different disease conditions, with higher levels found in the gut microbiota of patients with ulcerative colitis and irritable bowel syndrome than in that of healthy individuals (Lu et al., 2024). *Salmonella* is a common foodborne pathogenic bacterium in the intestine, widely present in nature, and can cause diseases such as typhoid fever, paratyphoid fever, and food poisoning (Lamichhane et al., 2024). In summary, compared with DM patients, those with DKD exhibited higher abundances of *Enterocloster*, *Shigella*, *Lachnoclostridium*, *Hallella*, and *Sellimonas*. These bacteria may play a significant role in the progression from DM to DKD.

Microbial and host-derived metabolites act as key “bridges” in host–microbiota crosstalk (Zhao et al., 2024). The metabolites implicated in DKD include phosphatidylcholine (Wang et al., 2023), SCFAs (Wang et al., 2025), and tryptophan-related metabolites (Corradi et al., 2024). In DKD, a decrease in SCFA-producing commensals has been reported, promoting inflammation and potentially accelerating disease progression, which is consistent with our data (Lan et al., 2023). Within the gut, tryptophan is metabolized to indole and related derivatives, including indoxyl sulfate and indole-3-acetic acid (IAA), which engage the aryl hydrocarbon receptor (AHR) to shape mucosal ecology and immunity, thereby influencing the gut–kidney axis (Caggiano et al., 2023).Compared with DM patients, those with DKD exhibited significant changes in microbial functions, including enrichment of pathways such as bacterial chemotaxis, phenylalanine, tyrosine, and tryptophan biosynthesis, *Escherichia coli* biofilm formation, flagellar assembly, and lipopolysaccharide (LPS) biosynthesis.

Previous research has demonstrated that, compared to control groups, elevated uraemic toxin levels are significantly positively correlated with the abundance of *Eggerthella* (Zhang et al., 2023; Zaki et al., 2025). In our study, we also observed an increase in *Eggerthella*, indicating that *Eggerthella* may play a crucial role in modulating pathways such as phenylalanine, tyrosine, and tryptophan biosynthesis during the transition from DM to DKD. LPS, a potent endotoxin found in the outer membrane of gram-negative bacteria, induces chronic inflammation. Gut dysbiosis and consequent increase in LPS levels may intensify the inflammatory response in patients with type 2 diabetes complicated by CKD (Salguero et al., 2019). Furthermore, we examined the relationships between the microbial communities, differential taxa, and clinical factors. Notably, taxa such as *Enterocloster*, *Limosilactobacillus*, *Thomasclavelia*, *Lachnoclostridium*, *Eggerthella*, *Sellimonas*, and *Staphylococcus* were significantly positively correlated with glycemic indices as well as with renal function parameters. Conversely, among the beneficial bacteria, except for *Faecalibacterium*, *Simiaoa*, and *Azospirillum*, the remaining seven significantly depleted taxa exhibited significant negative correlations with both glycemic and renal parameters. Bacteria in the gut may facilitate the progression from DM to DKD by modulating tryptophan metabolism, biofilm formation, and LPS synthesis.

Our functional profiling suggests enrichment of aromatic amino acid biosynthesis, biofilm formation, and LPS biosynthesis in DKD relative to DM. First, gut microbiota metabolized aromatic amino acids to produce uremic toxin precursors such as indoxyl sulfate and p-cresyl sulfate. Sulfation within the host completes toxin formation. These toxins act as AhR ligands, promoting inflammatory and fibrotic processes in renal and immune compartments (Xie et al., 2024; Yu et al., 2024). Second, LPS increases intestinal permeability by engaging TLR4 and driving NLRP3 inflammasome activation, thereby elevating circulating endotoxin levels and inducing mild inflammation associated with renal injury (Li et al., 2025). Third, biofilm formation stabilizes dysbiotic consortia and may facilitate endotoxin and uremic toxin persistence (Yang-Jensen et al., 2025).

This study has several limitations. First, the analyses are observational and lack mechanistic and causal validation, limiting definitive inferences about microbiota–DKD pathways. Second, our experiment did not include a long-term follow-up study. Third, this was a single-center study conducted exclusively in the beijing regions. In future work, we will recruit multi-regional, multicenter cohorts with longitudinal follow-up and progression endpoints, integrate multi-omics profiling, and directly quantify key metabolites to substantiate mechanistic links.

## Conclusions

This study conducted metagenomic analysis on fecal samples from patients with DKD, DM, and healthy controls. Our findings indicated that there was no significant difference in alpha diversity between DKD patients and healthy controls. However, the Simpson index in DKD patients was significantly higher than that in DM patients. In terms of beta diversity, significant differences were observed between DKD patients and healthy controls, while no significant differences were detected between DKD and DM patients. Regarding microbial composition, harmful bacteria such as *Mediterraneibacter*, *Enterocloster*, *Shigella*, and *Limosilactobacillus* were enriched in both the DKD and DM groups. Conversely, beneficial bacterial communities, including *Phocaeicola*, *Faecalibacterium*, *Lachnospira*, and *Agathobacter* were significantly enriched in healthy controls. Notably, the abundance of *Enterocloster*, *Shigella*, *Lachnoclostridium*, *Hallella*, and *Sellimonas* was higher in DKD patients than in DM patients, suggesting that these bacteria may play a role in the progression from DM to DKD. Functional analysis revealed that, compared with healthy controls, pathways such as galactose metabolism, pyruvate metabolism, and the phosphotransferase system (PTS) were significantly enriched in DKD patients, while carbon metabolism, galactose metabolism, pyruvate metabolism, and PTS functions were notably enriched in the DM patients. In addition, correlation analyses demonstrated that the bacteria significantly enriched in DKD and DM patients were positively associated with several clinical parameters, particularly HbA1c, fasting blood glucose, and UACR.

In summary, this study elucidates the distinct characteristics of the gut microbiota in DKD patients and highlights potential microbial markers involved in the progression from DM to DKD. Future investigations should further explore the interactions between microbial metabolites and the gut microbiota to deepen our understanding of their roles in DKD pathogenesis.

## Data Availability

The datasets presented in this study are available in the main article and **Supplementary Material**. For further inquiries, please contact the corresponding author directly.
