# Supplementary figures and images for: Characterization of gut microbiota in patients with diabetic kidney disease
Source: Front Cell Infect Microbiol. 2026 Feb 9;16:1713005. doi: 10.3389/fcimb.2026.1713005 (PMC12926362; doi:10.3389/fcimb.2026.1713005)

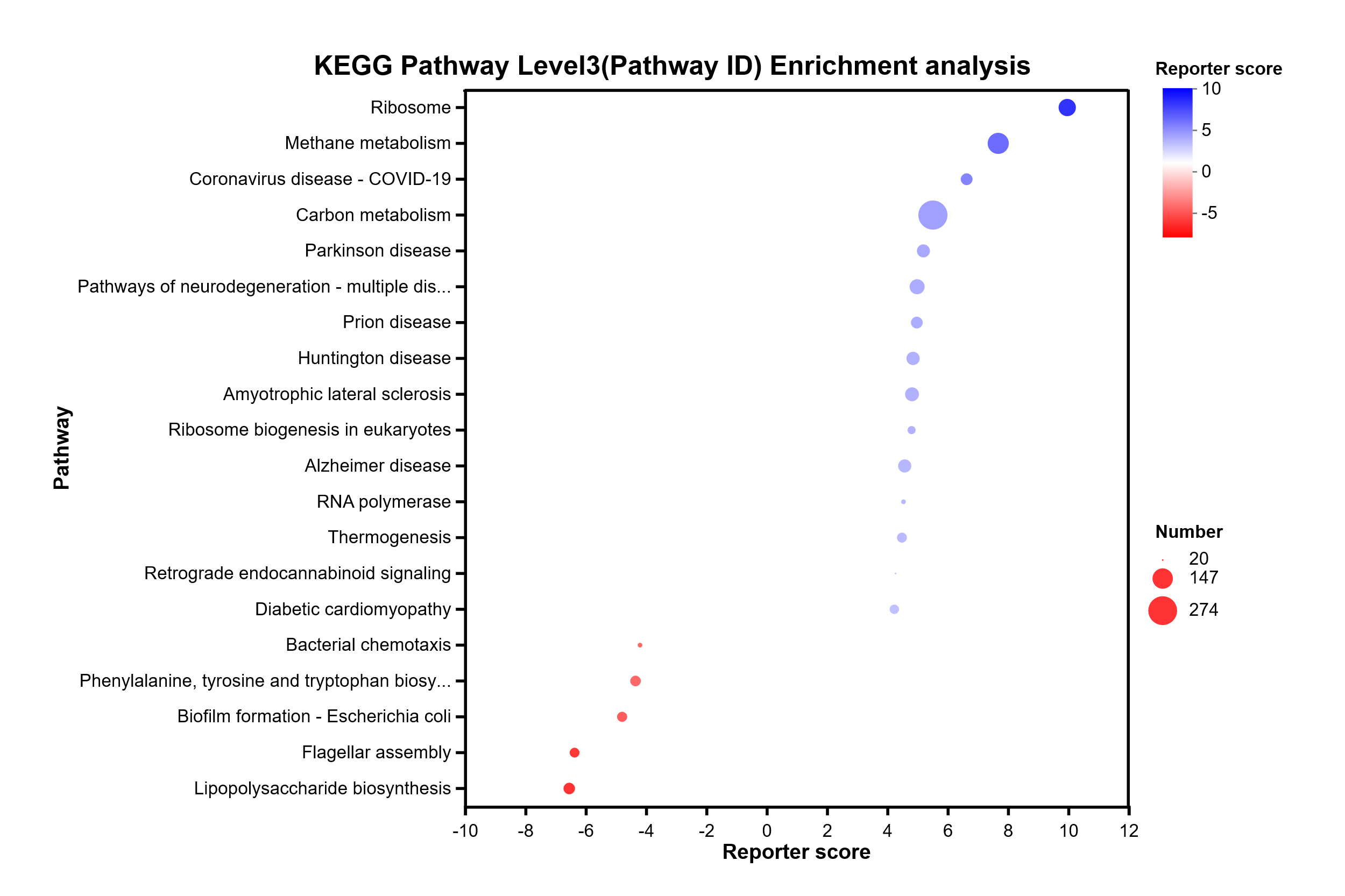


FIG. S1 KEGG Pathway Level3 (Pathway ID) Enrichment analysis (DKD vs DM)

Supplement: Supplementary file 1 [file DataSheet1.docx]
